# The Impact of IPTi and IPTc Interventions on Malaria Clinical Burden – *In Silico* Perspectives

**DOI:** 10.1371/journal.pone.0006627

**Published:** 2009-08-13

**Authors:** Ricardo Águas, José M. L. Lourenço, M. Gabriela M. Gomes, Lisa J. White

**Affiliations:** 1 Instituto Gulbenkian de Ciência, Oeiras, Portugal; 2 Centro de Matemática e Aplicações Fundamentais, Universidade de Lisboa, Lisboa, Portugal; 3 Mahidol-Oxford Tropical Medicine Research Unit (MORU) Faculty of Tropical Medicine Mahidol University, Bangkok, Thailand; London School of Hygiene & Tropical Medicine, United Kingdom

## Abstract

**Background:**

Clinical management of malaria is a major health issue in sub-Saharan Africa. New strategies based on intermittent preventive treatment (IPT) can tackle disease burden by simultaneously reducing frequency of infections and life-threatening illness in infants (IPTi) and children (IPTc), while allowing for immunity to build up. However, concerns as to whether immunity develops efficiently in treated individuals, and whether there is a rebound effect after treatment is halted, have made it imperative to define the effects that IPTi and IPTc exert on the clinical malaria scenario.

**Methods and Findings:**

Here, we simulate several schemes of intervention under different transmission settings, while varying immunity build up assumptions. Our model predicts that infection risk and effectiveness of acquisition of clinical immunity under prophylactic effect are associated to intervention impact during treatment and follow-up periods. These effects vary across regions of different endemicity and are highly correlated with the interplay between the timing of interventions in age and the age dependent risk of acquiring an infection. However, even when significant rebound effects are predicted to occur, the overall intervention impact is positive.

**Conclusions:**

IPTi is predicted to have minimal impact on the acquisition of clinical immunity, since it does not interfere with the occurrence of mild infections, thus failing to reduce the underlying force of infection. On the contrary, IPTc has a significant potential to reduce transmission, specifically in areas where it is already low to moderate.

## Introduction

Malaria exerts a huge morbidity and mortality toll on people in sub-Saharan Africa [1], [2]. This heavy burden raises the need to optimise control tools and devise appropriate intervention schemes. Several trials have tried to reduce the risk of acquisition of life threatening malaria infections through the use of prophylaxis [3]–[5]. In endemic settings, prophylaxis has been shown to protect children from episodes of malaria, anaemia and death [6]. Despite its beneficial impact, mass implementation of chemoprophylaxis raises concerns that need to be taken into serious consideration: (1) whether immunity in treated individuals develops as in untreated ones (whether there is a rebound effect); (2) spread of drug resistance; (3) costs; (4) logistic complexity.

New strategies designed to deal with such potential drawbacks have been put forward and into practice. Particularly, the administration of anti-malarial drugs to pregnant women through intermittent preventive treatment (IPTp), has been quite successful at protecting them from malaria and placental parasitaemia, and their newborns from malaria-associated low birth weight [7], [8]. Results encouraged the idealisation of new strategies that could revolutionise clinical management of malaria, by effectively targeting at-risk patients in endemic areas. Since in endemic areas incidence of clinical malaria is higher in young children [9]–[11] and pregnant women [12], the IPT strategy was later on expanded to infants [13]–[16] and children [17]–[19]. Intermittent preventive treatment consists of the administration of a therapeutic course of an anti-malarial drug at predetermined intervals, regardless of infection status [20], [21]. The purpose is to clear any current infection and prevent new ones. The intervals between doses are longer than the time to clear the drug from the bloodstream (although this would depend on the type of drug used), ideally allowing for infections between doses. The ultimate goal is, then, to simultaneously reduce frequency of infection and life-threatening illness, while allowing immunity to build up.

Because fewer doses are given compared to chemoprophylaxis, concerns about cost and drug resistance are significantly reduced. We will not dwell on how IPT might alter emergence and spread of drug resistance, as it has been studied elsewhere [22], [23]. Whereas in infants (IPTi) delivery is made during the routine vaccinations of the Expanded Programme on Immunization (EPI), for IPT in children (IPTc) drug delivery is not supported by any existing public health program, but rather relies on community volunteers. Nevertheless, a pilot study conducted in Senegal achieved over 80% IPTc coverage while using community volunteers for drug deployment [24], which attests the logistic feasibility of such an intervention.

The complexities of malaria epidemiology have huge implications for the unsettled question of whether immunity in treated individuals develops as in untreated ones, and a precise evaluation of IPT interventions efficacy in different endemic settings is required. It is important to define as clearly as possible the conditions under which the current conception of IPTi within the EPI delivery system, and IPTc administered to a specific age range of children, according to a particular schedule will be beneficial. We will explore the possible scenarios by simulating different schemes of intervention and transmission settings, relying on empirical data [15], [19] for model calibration.

The establishment of whether (and if so under which conditions) a rebound effect is observed is of major importance to advise control programmes being implemented in the field. So far, reports on outcomes of IPTi interventions have been inconclusive and contradictory to some extent. Whereas all studies show a great decrease (at least 20%) in the number of clinical cases during the treatment period [13]–[16], there is a noticeable disparity regarding follow up period results. Some studies report extended protection after treatment [15], while others claim an increase in clinical cases [13], [14], even as high as 100% more cases of severe malaria in the treated group [14]. Previous modelling work has predicted IPTi efficacy to be higher where IPTi coverage is greater, the health system treatment coverage lower, and for more efficacious and longer lasting drugs [25]. Also, IPTi impact on transmission intensity was estimated to be negligible [25]. The two models developed so far have suggested that there is increased susceptibility between doses and following the last dose, although these effects are outweighed by the overall intervention benefits [25], [26].

For IPTc, the scarce data that exists refers to studies conducted in seasonal transmission settings, in which reported efficacy reached as much as 90% [17]–[19]. No long term follow up data are available at the moment. An important question is then to assess the optimal timing for the administration of drug courses in areas where malaria transmission is highly seasonal.

## Methods

### Transmission model

We built a simple model to simulate IPT interventions in a given cohort. Overlaid on a basic topology describing the transmission of malaria in a given population [27], we apply an intervention strategy consisting of giving a varying number of therapeutic courses of an anti-malarial drug. The model is represented by the system of differential equations:
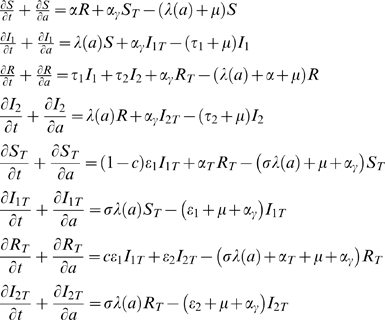
(1)where each variable represents the proportion of the population at any given age and time in eight epidemiological states: completely susceptible untreated (*S*) and treated (*S_T_*); clinical malaria resulting from infection in a completely susceptible untreated (*I_1_*) or treated individual (*I_1T_*); recovered with clinical natural immunity (*R*) or immunity acquired while treated (*R_T_*); and mild or asymptomatic infection resulting from exposure of recovered untreated (*I*_2_) or treated individuals (*I*_2*T*_). The force of infection is an age-dependent function defined previously in [27] as:
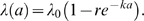
(2)


The function is strictly increasing with age, with a minimum *λ*_0_(1−*r*) (at age zero) converging asymptotically to *λ*_0_ as age increases. Parameter *k* determines how steeply the force of infection increases with age, and *r* controls the magnitude of that increase.

A summary measure of transmission is obtained by integrating the force of infection over age as:
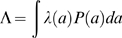
(3)where 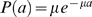 is the total population distributed over age and μ is the birth and death rate. One must bear in mind that this formulation is generic and should be adjusted for specific populations by using real population age profiles.

Adopting standard assumptions, *Λ* is proportional to the frequency of infectious individuals, the proportionality constant being the transmission coefficient, 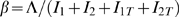
(4)


The force of infection can be seasonally forced by making *β* a time dependent variable. Adapting a standard sinusoidal function:
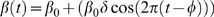
(5)where *β*_0_ is the baseline transmission coefficient, and *δ* is the amplitude, and *φ* the phase of the variation.

The boundary conditions for system (1) at age *a* = 0 are 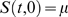 and 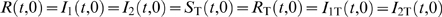. We used the escalator boxcar train (EBT) technique to model the dynamics in our age structured population. This is a numerical method used for physiologically structured population models [28].

The transmission parameter values for the non-treated classes were estimated from datasets from 8 regions in sub-Saharan Africa [27]. Treatment moves a proportion of individuals of each class, determined by the intervention programme coverage, *γ*, to the corresponding treated class, represented by subscript *T*. The dynamics of transmission in the treated compartments mimics that of the basic model, but is governed by different parameters representing the prophylactic effect of anti-malarial drugs, which wanes at a rate *α_γ_*. Rates of drug clearance and recovery from infection for the treated classes are taken from the literature [29]–[31]. Immunity in the treated classes also wanes, but not much is known about the dynamics of clinical protection loss. We have, thus, equalled it to the loss of immunity in the untreated classes. We introduce a parameter *c* to represent the proportion of clinical infections in treated individuals that acquire clinical immunity. We also introduce a parameter *σ* to account for the reduction in the risk of acquiring a new infection while treated.

**Figure 1:**
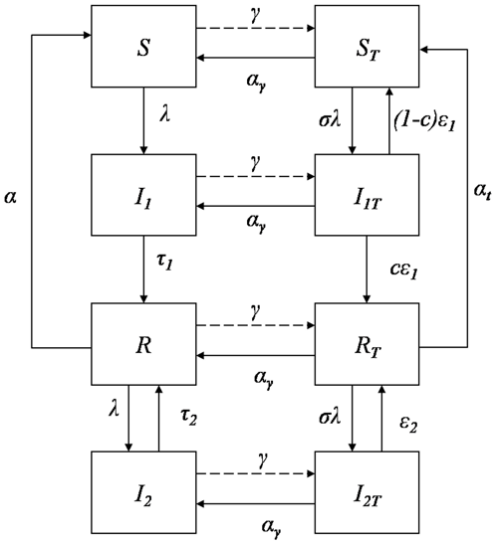
Model representing the dynamics of malaria transmission in a population under treatment. The model is an extension of the model in [27]. The compartments represent the following epidemiological classes: *S* = completely susceptible individuals, either newborns, or individuals that have lost protection conferred in *R*, or have cleared drugs from the bloodstream while in *S_T_*; *I*_1_ = clinical malaria resulting from an infection in a completely susceptible individual or after drug clearance from *I_1T_*; *R* = individuals that recovered from infections *I_1_* or *I_2_* or that have cleared the drugs from circulation while in *R_T_*, and are clinically immune, developing a mild form of disease if exposed. *I*_2_ = mild/asymptomatic disease resulting from exposure of recovered individuals or drug clearance while in *I_2T_*. *S_T_* = completely susceptible individuals that were treated, have lost immunity conferred in *R_T_*, or failed to build up their immunity after an infection. *I_1T_* = severe disease resulting from an infection in a treated susceptible individual, or treated while in *I_1_*. *R_T_* = individuals that recovered from infections *I_1T_* or *I_2T_* and acquired clinical protection, or where treated while in *R*. *I*_2T_ = mild/asymptomatic disease resulting from exposure of *R_T_* individuals or treated while in *I_2_*. The parameters are described in Table 1. A percentage of infants (according to programme coverage) depicted as *γ* is discretely moved to the corresponding treated classes, at specific ages.

The model dynamics are schematically represented in Figure 1 and the model parameters defined in Table 1.

**Table 1:** Model parameters.

Parameter	Definition	Value
*μ*	Birth and death rate	1/50 yrs^−1^
*τ_1_*	Rate of recovery from *I_1_* infections	14.12 yrs^−1^
*τ_2_*	Rate of recovery from *I_2_* infections	2.23 yrs^−1^
*α*	Rate of loss of acquired immunity in *R*	1.07 yrs^−1^
*γ*	Prophylaxis coverage	0.9
*η*	First line drug treatment coverage	0.24
*αγ*	Clearance rate of the drugs from the bloodstream	12 yrs^−1^
*αt*	Loss of immunity acquired while treated	1.07 yrs^−1^
*σ*	Reduced risk of infection while treated	Variable
*c*	Probability of clinical immunity acquisition	Variable
*ε_1_*	Recovery rate from *I_1T_* infections	120 yrs^−1^
*ε_1_*	Recovery rate from *I_2T_* infections	120 yrs^−1^
*β*	Transmission coefficient	Variable
*φ*	Phase of seasonal fluctuations	Variable
*δ*	Amplitude of seasonal fluctuations	0.5
*λ(a)*	Age dependent force of infection	Variable

### Modelling IPTi

To assert the benefits of intervening on a given endemic population, we first simulate our age structured model in equilibrium conditions to obtain the age profile of clinical disease prevalence without any intervention. We use that age profile as the initial condition for the simulation in which IPTi is implemented. We assume EPI coverage of 90% in concordance with the guidelines for the EPI initiative [32], and simulate a scenario of intervention at ages 2, 3 and 9 months for 10 years. We assumed that a percentage (*η*) of clinical malaria infections is undergoing first line drug treatment. We consider that 13% of clinical cases are severe [33] and that 50% of those receive appropriate first line treatment [25]. The remaining non-severe clinical malaria cases are assumed to be treated with a 20% probability [25]. The individuals undergoing first line treatment are precluded from receiving any IPTi dose. Intervention works by, at each instant in time, moving individuals in untreated classes of cohorts that have just entered an age class corresponding to any age of intervention, to the corresponding treated classes, according to their respective IPT coverage rate. Intervention outcome is measured in terms of impact, defined as the percentage reduction in infection or disease caused by an intervention in a trial group (simulated intervention) compared to a control group (simulation without any intervention).

### Modelling IPTc

Simulation of IPTc interventions is similar to that of IPTi, except intervention is not continuous in time, but rather for a specific age range. Specifically, interventions occur at predetermined instances over a 1 year period, targeting a given age range. We decided to mimic one study conducted in primary schools in Kenya, in which children aged 5–18 years received 3 doses of anti-malarial drugs at 4 months intervals [19], since this is the study that comprised the largest number of people, with the largest age span, over the greatest period of time. We consider these study characteristics as ideal premises to compare our model with data and test the dynamics between pulses of drug administration, as well as the long term impact of IPTc. We expanded our analysis to a second study which focuses on the administration of IPTc targeting the high transmission season, in a markedly seasonal setting [34]. Here, children aged 2 months to 5 years of age receive a monthly course of drugs, for 3 months. This is an ideal setup to investigate the importance of seasonality and drug administration timing on intervention outcome.

### Implementing seasonality

The general time dependent transmission function was adjusted to the two settings simulated here [19], [34]. Whereas in the Kenyan study site transmission is intense and perennial, with two seasonal peaks (March-May and November-December [19]); in the Senegalese region there is a single high transmission season from August to October [34]. The seasonal transmission coefficients for the Kenyan site in study [19] (B_K_) and for the Senegalese site in [39] (B_S_) were then defined as:
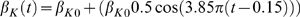
(6)

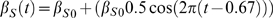
(7)


## Results

We will present the results either in the form of age profiles retrieved at specific points in time, or time plots, where each point represents the integral of the corresponding age profile. We will convey the malaria scenario in the simulated population as proportions of clinical malaria and parasite prevalence. The former is equal to the proportion of people with clinical malaria in the overall population, in other words, the prevalence of clinical malaria, while the later is the proportion of people infected with a malaria parasite in the population, regardless of symptoms.

For the sake of simplicity we have established a nomenclature to define the transmission levels associated to the simulations. The most reliable measure of transmission on a given region is the overall parasite prevalence registered at a given time. We have, then, defined 3 classes of transmission according to parasite prevalence. Henceforth, we will refer to as low transmission settings, those regions where a cross-sectional survey would detect parasites is less than 10% of the individuals. If that value is within the 11–50% range, the region is defined as an intermediate transmission setting, while parasite prevalence above 50% would categorize the region as a high transmission setting.

### IPTi

**Figure 2:**
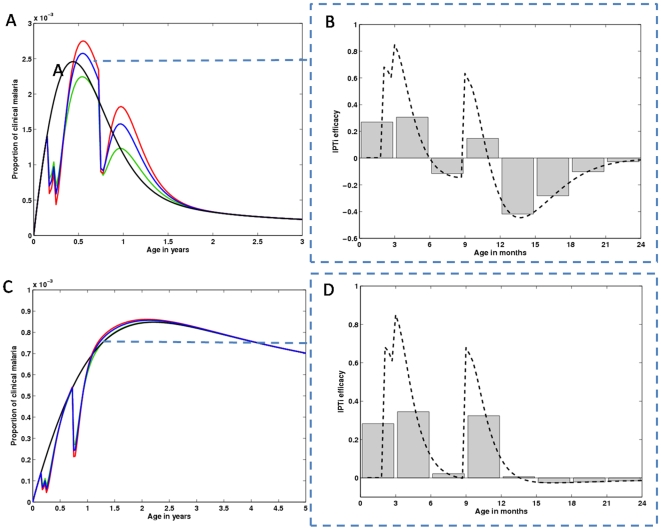
IPTi impact on clinical malaria age profiles. Analysis of the outcome of applying prophylactics at 2, 3 and 9 months of age, for 10 years, in terms of age profile of clinical disease prevalence and intervention efficacy. Age profiles of populations under IPTi are compared with populations without intervention, in equilibrium conditions (black line). (A) Simulations assuming different combinations of values for *c* and *σ*, under intense malaria transmission. The values for these 2 parameters are equal for each curve, ranging from 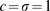 (green line) to 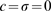 (red line). The blue line represents the intermediate combination, 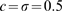. (B) The dashed line represents the age instantaneous intervention efficacy for the blue curve scenario in (A). The grey bars illustrate efficacy over a 3 months range. (C) Represents the same as in (A), but for a intermediate transmission setting. (D) The dashed line represents the age instantaneous intervention efficacy for the blue curve scenario in (C). The grey bars illustrate efficacy over a 3 months period.

Figures 2A and 2C investigate how the probability of acquiring clinical immunity when infected while in the *S_T_* class, *c*, and the reduced risk of acquiring an infection while treated, *σ*, modulate the age profiles of clinical malaria. We show the age profiles for the parameter combinations that result in the best (green line) and worst (red line) intervention impacts, as well as a parameter combination assuming an empirical estimate for *σ* [35]–[37] and a conservative guess for *c* (blue line). In Figure 2A, we use a value for the force of infection corresponding to a high transmission setting (in this case parasite prevalence if around 90%). We observe that, if immunity is efficiently acquired upon clinical infection,*c* = 1, and the drug does not reduce the risk of having a malaria infection, 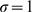 (green line), the predicted rebound effect is minimised. For intermediate levels, 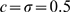, of these parameters (blue line), the model suggests that rebound becomes significant after the first year of life. This effect is exacerbated when treatment prevents infection from occurring and clinical immunity is not built up due to prophylaxis, 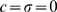 (red line). From the green, 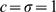, to the red line, 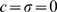, the proportion of cases predicted to be prevented up to age 10 decreases from 12.8% to 0.9%, being 5.6% for the blue line, 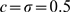 (Table 2). Generally, these results suggest that there is an evident beneficial effect at the ages targeted by treatment, regardless of parameter values. More importantly, there is a noticeable rebound effect, meaning that the treated group is at increased risk of having a clinical malaria episode, after the last dose of treatment.

**Table 2:** Intervention impact below 10 years of age, for different values of *c* and σ, under high transmission.

		*c Probability of clinical immunity acquisition*					
		0		0.25	0.5	0.75	1
***σ*** ** Reduced risk of infection**	**1**		0.0091	0.0458	0.0773	0.1046	0.1285
	**0.75**		0.0090	0.0397	0.0669	0.0911	0.1128
	**0.5**		0.0090	0.0334	0.0557	0.0762	0.0951
	**0.25**		0.0089	0.0267	0.0435	0.0595	0.0747
	**0**		0.0089	0.0196	0.0302	0.0407	0.0511

Impact is measured as the percentage reduction in malaria clinical cases caused by IPT in a trial group compared to a population without intervention.

Figure 2C shows the simulated effectiveness of anti-malarial drugs during treatment and follow-up periods, in an intermediate transmission setting. The results described for Figure 2A are maintained for lower transmission, except that both intervention impact and rebound effect are at a smaller scale. This translates into a prophylactic efficacy from ages 0 to 10 years old that range from 1.04% to 1.6%, depending on the values for *σ* and *c* (Table 3).

**Table 3:** Intervention impact below 10 years of age, for different values of *c* and *σ*, under intermediate transmission.

		*c Probability of clinical immunity acquisition*					
		0		0.25	0.5	0.75	1
***σ*** ** Reduced risk of infection**	**1**		0.0104	0.0120	0.0135	0.0150	0.0164
	**0.75**		0.0106	0.0119	0.0131	0.0144	0.0156
	**0.5**		0.0108	0.0118	0.0128	0.0138	0.0148
	**0.25**		0.0110	0.0117	0.0124	0.0132	0.0139
	**0**		0.0111	0.0116	0.0121	0.0126	0.0130

Figure 2B,D displays in greater detail how the simulated IPTi protective efficacy changes over age, when 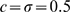. The highest protective effects are always at the ages at which prophylaxis is administered, and this protection gradually declines as drug effect wanes. The larger rebound effect is expected to happen during the first trimester of the second year of life, although for higher values of both *σ* and *c*, a rebound might occur between the second and third prophylactic treatments. The extent of the rebound period is highly sensitive to the duration of drug effect.

**Figure 3:**
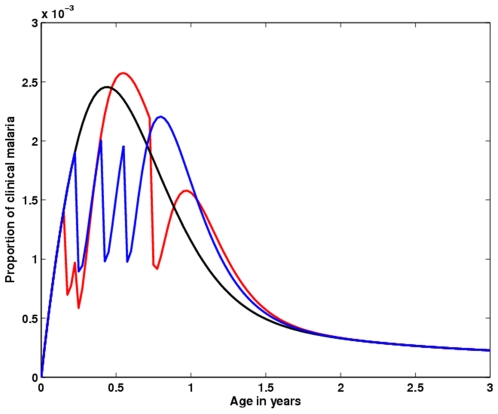
Targeted interventions according to endemic level. The IPTi intervention outcome (red line) is compared with a population without intervention in equilibrium conditions (black line), and a tailored schedule for the administration of anti-malarial drugs (blue line). These simulations are performed for a high transmission transmission setting.

In Figure 3 we analyse the impact of IPTi at a specific point of the transmission spectrum, focusing on the importance of tailored interventions. In these simulations we assumed that, while treated, the risk of infection upon challenge, and the chance to build up clinical immunity upon infection is 0.5. We present in red an IPTi schedule concomitant with the EPI vaccination ages, and in blue the simulated intervention scenario that resulted in higher impact in terms of proportion of clinical cases prevented. The predicted optimal schedule for a three-dose intervention over the first year of life for this specific transmission setting is to give prophylactic treatment at 3, 5 and 7 months of age. Whereas in intermediate transmission areas, targeted interventions seem to harness little benefit (not shown), in high transmission regions a tailored schedule may be responsible for the prevention of 8.3% of cases of clinical malaria over all age classes, which contrasts with the 5.6% obtained under the EPI schedule.

**Figure 4:**
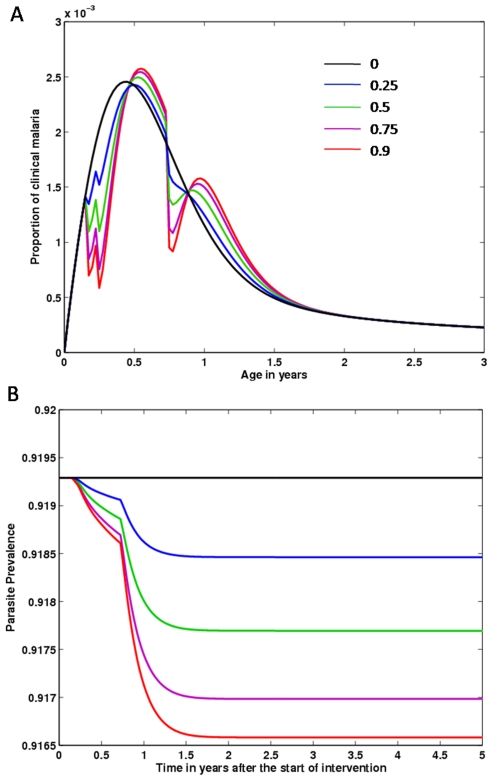
Intervention impact on clinical malaria and overall parasite prevalence. (A) IPTi impact on clinical malaria, assuming several values for intervention coverage, as specified in the figure legend. (B) Time dynamics of the proportion of people infected with malaria, assuming different IPTi coverage rates as in (A).

Figure 4A illustrates how program coverage is a critical determinant of intervention outcome. Detailed analysis revealed that the higher the proportion of the population under treatment, the better the overall intervention impact is (Table 4). Despite there being a higher rebound effect for higher IPTi coverage, the largely lowered burden exerted on the first year of age renders these interventions more effective against clinical malaria. High coverage also achieves better impact on parasite prevalence, although the actual reduction in the proportion of infectious people is minimal (Figure 4B). These results have been proposed by other modelling studies [25], [26].

**Table 4:** IPTi impact below 10 years of age, in a high transmission region, under different intervention coverage.

IPTi coverage	Impact <1 yr of age	Impact >1 yr of age	Overall impact
0.9	0.1998	−0.0869	0.0557
0.75	0.1697	−0.0730	0.0477
0.5	0.1164	−0.0494	0.0331
0.25	0.0597	−0.0250	0.0171

### IPTc

**Figure 5:**
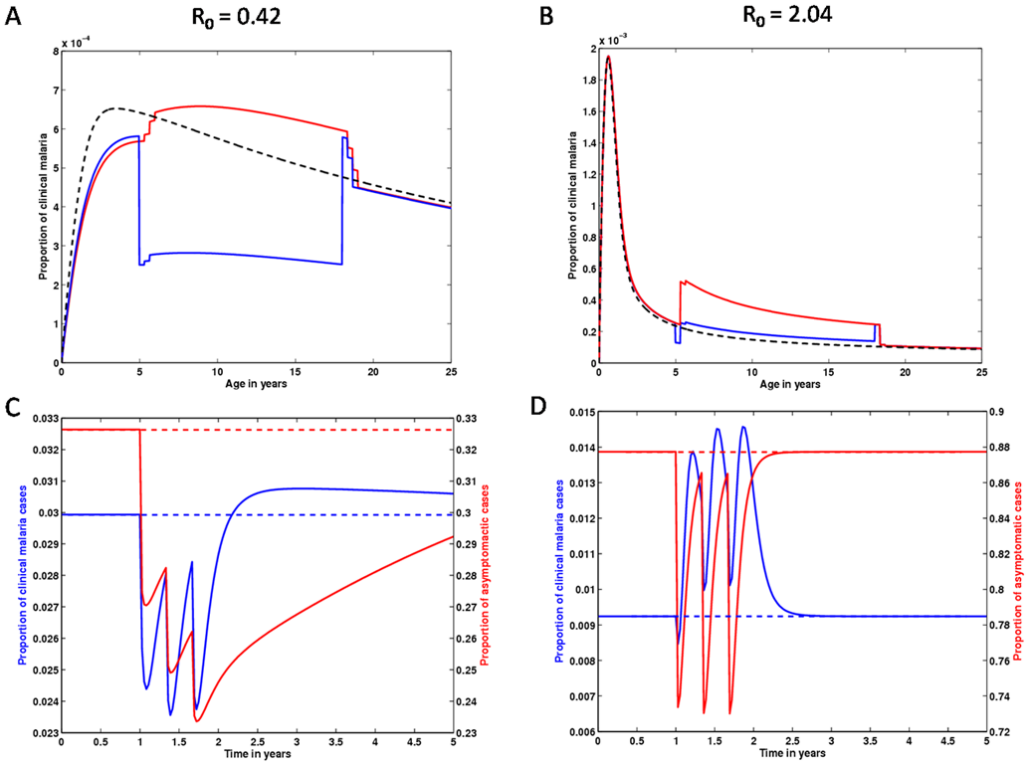
Impact of an IPTc strategy apllied in 5 to 18 years old children on clinical malaria age profiles, and over time. Age profiles of populations under an IPTc intervention calibrated from data in Clarke et al. [19] are compared with populations without intervention, represented by black lines. (A) Clinical malaria age profiles, retrieved immediately after the third dose of treatment (blue line), and 4 months after the administration of that course of drug (red line). (B) Represents the same as (A), but for a high transmission setting. (C), (D) Time dynamics of IPTc impact over all age classes in mild and intense malaria transmission areas, respectively. Intervention (starting in year 1) shapes the dynamics of both clinical (blue line) and asymptomatic/mild (red line) malaria. The dashed lines represent unperturbed equilibria.

IPTc intervention significantly disturbs the age dependent risk of acquiring a malaria infection, which can be translated into age profiles of clinical episodes as those in Figures 5–7.

**Figure 6 pone-0006627-g006:**
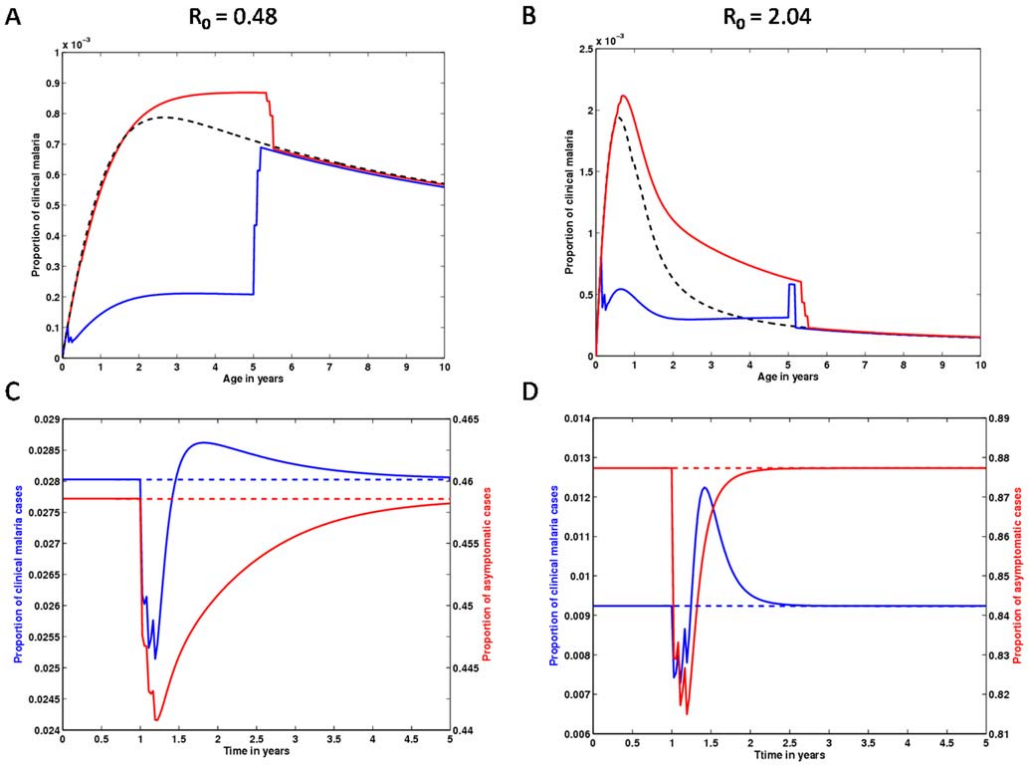
Impact of an IPTc strategy applied in 2 months to 5 years old children on clinical malaria age profiles, and over time. The same as in Figure 5 but calibrated according to Cisse et al [34].

**Figure 7:**
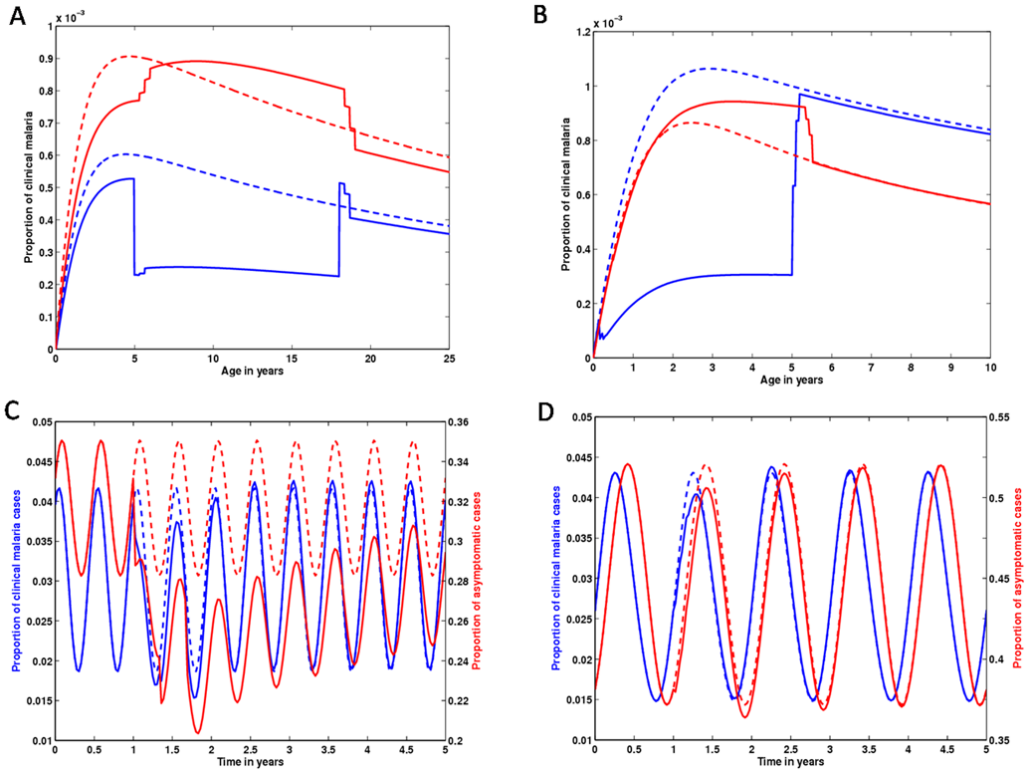
IPTc impact in seasonal settings. We mimic two studies conducted in regions with different seasonal transmission patterns [19], [34]. (A) Clinical malaria age profiles, immediately after the administration of the third dose of treatment (blue line), and 4 months after that (red line), for the study in [19]. The dashed lines represent the age profiles retrieved under no treatment, for each of the mentioned times points. Differences in the dashed lines refer to oscillations in transmission from one time point to the other. Intervention impact is measured as the difference between dashed and full lines of the same color (C) Time dynamics of clinical (blue line) and asymptomatic malaria (red line) in a population in which IPTc was implemented beginning in year 1 (full lines), following the schedule in [19], and in a population under no treatment (dashed lines). Replicating (A) and (C), whilst using the characteristics of IPTc implementation in [34] and the seasonal transmission fluctuations in [42], we get (B) and (D), respectively.

In Figure 5A,C, we replicate the IPTc study carried out in in western Kenya, by Clarke et al. [19]. We use the same intervention schedule and a force of infection that reproduces the observed pre-intervention parasite prevalence (close to 40%). The model was able to reproduce this study's results in terms of the effect on the parasite prevalence (PP) in children aged 5 to 18 years. The study reports 89% (73%–95%) reduction in PP, while we obtain impacts ranging from 81.2% to 84.9% depending on the values for *c* and *σ*. IPTc can be extremely efficient in lowering the burden of clinical malaria in children (blue line in panel A), albeit at the cost of a rebound effect just after a few months (red line in panel A). The implementation of the same intervention in high transmission regions (parasite prevalence above 80%) (Figure 5B,D), indicates much lower efficacy in protecting against clinical episodes (blue line in panel B), but significant rebound in the age range under treatment (red line in panel B).

The dynamics of the impact of IPTc on clinical malaria age profiles is shown in the video S1 while Figure 5 shows selected profiles in age and time. In Figure 5A,B, we plot the simulated age profile of a population where all children between the ages of 5 and 18 years have just received their third dose of prophylaxis (blue line), and the corresponding age profiles four months after that dose (red line) and previous to the start of intervention (black line).

In Figure 5C,D, we visualize the time dynamics of both clinical and asymptomatic malaria infections by integrating the age profiles (from age 0 to 100 years) at each point in time, allowing us to understand the intervention induced disturbance over the time dynamics of malaria infections (both clinical and asymptomatic). As suggested by panels A and B, the overall impact of IPTc should be greater in intermediate transmission areas (Figure 5C) than in high transmission ones (Figure 5D). Figure 5C shows how protection against malarial infections is expected to be sustained for more than 3 years following a one year IPTc program (red line). However, this comes at the expense of a small increase in the number of clinical episodes (blue line). In high transmission regions, IPTc exerts a small pressure on the overall dynamics and the pre-intervention scenario is foressen to be restored in the year following intervention conclusion (Figure 5D).

We also simulated the study conducted in Senegal by Cisse et al [34], by using the adopted intervention schedule and calibrating the force of infection according to the observed pre-intervention parasite prevalence (Figure 6A,C). The procedure is replicated for a higher transmission setting (Figure 6B,D). Overall, the figure displays the same general results as those found in Figure 5, the difference being that the impact of IPTc on overall malaria transmission is at a smaller scale in the Senegalese study. The difference advents from the broader age range covered by IPTc in the Kenyan study [19].

In Figure 7A,C and 7B,D, we explore the effect of seasonality on the IPTc interventions performed in Kenya [19] (previously assessed in Figure 5) and Senegal [34] (previously assessed in Figure 6), respectively. This first exploration suggests that the effect of seasonality is quantitative, while the qualitative results remain unchanged. In Figure A,B, the model predictions indicate a very effective impact of IPTc over the clinical age profiles immediately after administration of a course of drugs, and an increase in risk following treatment. This qualitative behaviour is maintained regardless of the age range covered by IPTc, seasonal fluctuations in transmission, and transmission intensity. Again, we expect a more pronounced impact on malaria transmission in the Kenyan study, which encompasses a larger age range under prophylaxis. Although the time dynamics in Figures 5C and 6C are a good approximation for the average behaviour of what is observed in Figure 7C,D, this should be regarded as an initial insight and the study should be followed by a systematic analysis of varying intervention schedules. In particular, when the dynamics occurs near elimination thresholds, seasonality may be instrumental.

The seasonal model presented here is a valuable tool for the design of intervention schedules tailored to specific regions. A systematic analysis of this aspect is too extensive to be included in the present study.

## Discussion

Intermittent preventive treatment (IPT) is a very promising approach to the management of clinical malaria burden in infants and children. However, concerns as to whether immunity is being allowed to build up during treatment, or if disease risk is actually just being postponed to later ages (rebound effect), persist.

Although it is well established that parasites are rapidly cleared from the bloodstream after administration of anti-malarial drugs [29], [30], reports on the prophylactic effect of SP (drug of choice for both IPTc and IPTi) against a new infection refer mainly to malaria in pregnancy [35]–[37], pointing to a 30% to 60% reduced risk of infection. While data on how prophylaxis affects the probability of functional acquisition of immunity are inconclusive, this can be explored using mathematical models. To investigate the chances of immunity build up in individuals who have received a drug dose, we introduce to out model parameter, *c*, representing the probability of clinical immunity acquisition.

In the ideal scenario, where immunity is allowed to build up during treatment as efficiently as if the individual goes through a full length of infection (*c* = 1), and the risk of infection is not reduced by treatment (*σ* = 1), the modelled rebound effect is minimised. If immunity is not built up efficiently (*c*<1), the proportion of individuals still susceptible to clinical malaria after the last dose of prophylactics may be large, thus, causing a rebound effect in later ages. If the risk of infection is reduced by treatment (*σ*<1), boosting of immunity is less frequent and there is a greater risk of returning to the non-immune compartment. Intervention outcomes in ages following the last drug dose schedule are highly dependent on transmission levels and on how treatment affects infection dynamics, mainly the time for which the drug effect lasts, the drug induced decreased risk of acquiring an infection, and the ability to acquire functional clinical immunity while treated.

The model then suggests that the greater the risk of acquiring an infection while treated, and the more that infection resembles a natural one (in terms of inducing clinical immunity), the better the outcome of intervention in terms of effectiveness is, during both treatment and follow-up periods. Even when there is a noticeable rebound effect (such as when *c* = 0and 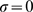), the benefits of implementing IPTi in infants are predicted to surpass the drawbacks of having more cases in older children (Tables 2, 3). This has been proposed by previous modelling studies [25], [26].

The simulated dynamics of age dependent efficacy of IPTi interventions are qualitatively equivalent in high transmission and intermediate transmission areas, albeit less pronounced in the later. In high transmission areas, incidence of clinical malaria is higher in young children [9]–[11] and pregnant women [12]. In these regions, the ages at which clinical malaria peaks coincide with the interval at which interventions are being made, which translates into a great effectiveness of intervention, although rebound may be present (Figure 2B). The model predicts that the number of cases prevented by intervening is greater than the excess of cases after intervention, giving an overall impact in clinical cases (across all age classes) ranging from 0.4% to 6.8%. In intermediate transmission areas, where occurrence of clinical cases is frequent in later ages [38], and where the average age at infection is much higher than the age at which prophylaxis is administered, the overall benefits of IPTi are almost negligible, and the rebound effect is not significant either (Figure 2D). The first year of age represents a small fraction of the cases of clinical malaria, which implies that an intervention such as IPTi has a small impact. The same relationship between transmission intensity and IPTi efficacy has been noted in [25].

Furthermore, we notice that, although administration of IPTi along with the EPI schedule facilitates logistics, the results using this schedule are far from being optimal in every region. To maximise the number of cases prevented by intervention at each age, the schedule of intervention should be tailored to each region according to their specific transmission levels. In intermediate transmission areas, IPTi should be most effective if the schedule is extended into early childhood. In high transmission regions a tailored schedule very similar to EPI may be responsible for the prevention of 2.7% more cases when compared to the typical IPTi schedule.

The relationship between endemicity and intervention efficacy is opposite when comparing IPTi and IPTc. Intermediate transmission regions are highly sensitive to IPTc, in contrast to the robustness of high transmission regions. Since intervention affects children and toddlers, the age range under treatment coincides with an age of high risk of acquisition of malaria when transmission is low to moderate. Our results suggest that IPTc has a major impact on malaria transmission during the intervention period in intermediate transmission areas, as reflected by the reduction in clinical cases, and more significantly by the large decrease in asymptomatic cases. A rebound of marginal magnitude in clinical cases is expected to occur less than one year after the last dose of SP.

Since IPTc intervention is discrete in time, it allows for a period of time between doses when individuals are not protected by prophylaxis, since SP is cleared from the blood stream in about one month. The large age range tested in [19] and simulated here ensures that efficacy is maximised, and the four month hiatus between courses of drugs ensures minimal rebound, since clinical immunity can be boosted by natural infections during this period. The study in [34], while affecting a smaller age range comprised of lower ages, used a monthly schedule of drug administration for a period of 3 months. This schedule is particularly efficient in tackling the clinical malaria burden in areas where transmission mainly occurs in a short period of the year. If transmission is sustained for a longer period, the time interval between doses should be increased.

IPT interventions revolve around the idea of simultaneously reducing the frequency of infection and life-threatening illness in infants or children, while allowing immunity to build up. We varied intervention coverage to explore the impact of IPTi on malaria transmission in a highly endemic region. Our model finds that high coverage has better results as to what concerns overall parasite prevalence, although the actual reduction in the proportion of infectious people is minimal. This contrasts with the effects IPTc displays over parasite prevalence. The model suggests that IPTc is more effective than IPTi in reducing the number of malaria cases, particularly in intermediate transmission areas.

Here we present a simplistic description of malaria transmission that does not encompass features such as heterogeneity (both at the level of host contact patterns and susceptibility) and stochasticity. Rather, we focus on the essential mechanisms which characterise malaria transmission on a population of individuals which exhibit identical behaviour. More detailed models which also simulate IPTi interventions [25], while discriminating transmission processes at the individual level and allowing for stochasticity, have obtained results which are strikingly similar to those presented here. Indeed, stochasticity is only a concern when dealing with very small numbers, such as in elimination scenarios. We recognise, however, that the introduction of heterogeneity and superinfection should increase the prevalence observed in the model, inducing some resistance to perturbations in transmission.

The baseline transmission model presented here was fully parameterised using datasets from several Sub-Saharan settings, encompassing a broad range of transmission intensities, and its parameters were subject to an extensive sensitivity analysis in [27]. The parameters defining the pharmacokinetics and pharmacodynamics are fairly well understood [29]–[31]. What is not known, however, is how drug dynamics and kinetics affect inherent immune and transmission processes. The rate at which drug effectiveness wanes is crucial in determining the extent of both protection against clinical episodes during the first year of life, and duration of rebound effect. Long lasting drugs confer extra efficacy to IPT strategies, although rebound becomes much more significant. IPT should also use a different combination of drugs compared with what is used for treatment of clinical cases, in order to minimize drug resistance issues. The exact value for the parameter defining how immunity wanes in treated classes is unknown. This parameter, however, has a small role to play, since the rate at which the drugs are cleared from the bloodstream is much faster than the rate of loss of clinical immunity. Implementation of large scale therapeutic interventions must be accompanied by careful assessment of the existing levels of drug resistance. IPTi yields very little impact on the overall transmission of malaria in endemic regions and is, therefore, unlikely to affect the spread of drug resistance. The situation is less straightforward for IPTc. Since this strategy is able to disturb malaria transmission to some extent, it is more likely to influence the dissemination of drug resistance, an aspect that deserves careful investigation.

Careful evaluation of the transmission characteristics of the areas under study can be extremely useful in deciding which program will be the most beneficial for each specific region. It has been observed that P. falciparum transmission is decreasing in some settings in sub-Saharan Africa [39], [40]. Under circumstances of falling malaria incidence, IPTi might become less efficacious and IPTc might be a good strategy to consider. Synergies between these and other control measures can also have a critical importance in determining the potential impact of an integrated intervention. This is particularly importance since interventions integrating preventative methods may overcome the rebounds predicted for single-intervention strategies [41]. These might be especially useful not only for malaria control but also for end phase elimination scenarios, where IPTc seems to be a good candidate as a resurgence precluding strategy, due to its transmission lowering potential.

## Supporting Information

Video S1 Dynamics of the impact of IPTc on clinical malaria age profiles
(0.25 MB AVI)
